# Sulfomethylation reactivity enhanced the Fenton oxidation pretreatment of bamboo residues for enzymatic digestibility and ethanol production

**DOI:** 10.3389/fbioe.2024.1344964

**Published:** 2024-01-26

**Authors:** Zhaoming Liu, Min Zhang, Qinpei Hou, Zhengjun Shi, Haiyan Yang, Dawei Wang, Jing Yang

**Affiliations:** ^1^ School of Materials and Chemical Engineering, Southwest Forestry University, Kunming, China; ^2^ Key Laboratory for Forest Resources Conservation and Utilization in the Southwest Mountains of China, Ministry of Education, Southwest Forestry University, Kunming, China

**Keywords:** bamboo residues, sulfomethylation, Fenton oxidation reaction, hydrophilicity, enzymatic saccharification, ethanol production

## Abstract

Bamboo is considered a renewable energy bioresource for solving the energy crisis and climate change. *Dendrocalamus branddisii* (DB) was first subjected to sulfomethylation reaction at 95°C for 3 h, followed by Fenton oxidation pretreatment at 22°C for 24 h. The synergistic effect of combined pretreatment dramatically improved enzymatic digestibility efficiency, with maximum yield of glucose and ethanol content of 71.11% and 16.47 g/L, respectively, increased by 4.7 and 6.11 time comparing with the single Fenton oxidation pretreatment. It was found that the hydrophobicity of substrate, content of surface lignin, degree of polymerization, and specific surface area have significant effects on the increase of enzymatic saccharification efficiency. It also revealed that sulfomethylation pre-extraction can improve the hydrophilicity of lignin, leading to the lignin dissolution, which was beneficial for subsequent Fenton pretreatment of bamboo biomass. This work provides some reference for Fenton oxidation pretreatment of bamboo biomass, which can not only promote the utilization of bamboo in southwest China, but also enhances the Fenton reaction in the bamboo biorefinery.

## 1 Introduction

Bamboo has extraordinary growth rate, high productivity and high polysaccharide content, making it an important renewable forest resource for producing biomass-derived products (Chaowana, 2013; Ouyang et al., 2022). Approximately 260 billion tons of bamboo processing residues are generated when bamboo is used to produce crafts, furniture and artificial board (Ouyang et al., 2022). The main components of residues are cellulose, hemicelluloses and lignin, of which carbohydrates can be converted into sugar-based compounds, further fermented into ethanol. However, the coating of hemicellulose and lignin on cellulose, the high level of lignin and crystalline structure of cellulose hindered the bioconversion efficiency of bamboo fiber into biochemicals and biofuels.

Pretreatment technology was crucial to overcome the recalcitrance of biomass and destroy the complex arrangement of native biopolymers (Sabogal-Otálora et al., 2022). Most chemical and physicochemical pretreatment required high energy inputs and harsh conditions, such as high temperature and extreme pH, which often lead to produce some toxic wastes, and these processes have high requirements to reactor and operational conditions (Kumari and Singh, 2018; Satari, et al., 2019). Fenton oxidation pretreatments (FOP), which simulates the degradation process of lignocellulosic biomass by white rot fungi, have been proven be a promising and low-cost method to destroy the recalcitrant structures of plant cell walls for biomass fractionation (Ray et al., 2010; Zhang et al., 2023). When Fe^2+^ catalyzes the decomposition of H_2_O_2_ under acidic conditions, it can product two types of hydroxyl radicals (HO· and HOO·) with high oxidative strength, which attack lignin more effectively than hydrogen peroxide. It has the advantages of simple operating conditions and short reaction time, in comparison with biological pretreatment (Kato et al., 2014; Jung et al., 2015).

However, it found that the delignification rate of Fenton oxidation pretreatment decreases over time (Bhattarai et al., 2015; Davaritouchaee et al., 2019), due to the very short half-life of HO· and HOO (Kato et al., 2014), which stops the transportation of reactive oxygen species to the cell wall. Jung et al. (2015) and He et al. (2015) reported that single Fenton pretreatment consumed a longer reaction time, but cannot result in the satisfactory results during the lignocellulosic biomass fractionation. Our previous work used Fenton reagent to treat four bamboo residues at 22°C for 24 h, and xylan removal of 26.72% and delignification of 24% were detected (Zhang et al., 2023). Most of Fenton oxidation pretreatments focused more on how to generate more free radicals for improving Fenton reaction performance of lignocellulose (Yu et al., 2017). But this means that a large amounts of reagents input (Fe^2+^ and H_2_O_2_) were consumed, which has a negative impact on industrial applications. In addition, the high degree lignification of bamboo allows hydroxyl radicals to only act on the surface of bamboo without invading the interior, thus resulting in less damage to the lignin-carbohydrate complex structure. Therefore, it is very urgent to find a method that can further enhance and activate the reactivity of Fenton oxidation pretreatments for bamboo fractionation. Sulfomethylation can improve the hydrophilicity of lignin under mild conditions, and was used in the pulping and production of lignosulfonates (Kazzaz et al., 2019; Zhang et al., 2019). The improvement of water-solubility in the substrate can promote subsequent modification, through the enhancement of phase transfer ability (Demuner et al., 2021). Therefore, we proposed that the sulfomethylation can be used to promote the reactivity of FOP by improving the hydrophilicity and phase transition ability of bamboo residues, which resulted in the enhancement of bamboo fractionation performance.

In this study, the combined pretreatment of sulfomethylation and Fenton oxidation reaction was explored to enhance the enzymatic saccharification efficiency of bamboo residues. The effects of these pretreated sample on sugars and ethanol production were evaluated and compared. In order to reveal the enhancement mechanism, the degradation of bamboo cell wall components and the structural characterization of substrates before and after pretreatment was studied.

## 2 Materials and methods

### 2.1 Materials and enzymes

Bamboo residues, *Dendrocalamus branddisii* (DB), were harvested from Yunnan Province, China. The DB was air-dried and ground using a high-speed disintegrator to obtain 40–60 mesh fractions. The filter paper activity of cellulase preparations (Cellulase CTec2) was 200 FPU/mL; and protein content of cellulase C2730 was 41 mg/mL, which were received from Shanghai Sigma Life Science Company.

### 2.2 Sequential sulfomethylation and Fenton oxidation pretreatment of D. branddisii

Twenty grams of bamboo residues were mixed with 2% (w/w) NaOH and 3% (v/v) formaldehyde solution, at a solid-liquid ratio of 1:15 (w/v) and 70°C for 1 h. Then added 20 g/L sodium sulfite to the mixture, treated it at 95°C for 3 h, filtered and washed the solid fraction three times with excess hot water and named SP-DB.

Fenton oxidation pretreatment was performed on SP-DB at a solid-liquid ratio of 1:20 (w/v) using FeSO_4_ • 7H_2_O of 16 mmol/L and 30% H_2_O_2_ of 4 mL. The mixture was treated at 22°C and 80 rpm for 24 h. Adjusted the pH of the reaction mixture to 3.0 using sulfuric acid. The whole slurry was collected and centrifuged, and the solid residues were washed with tap water until neutral pH. The single Fenton pretreated DB and the combined sulfomethylation and Fenton oxidation pretreated DB were denoted as FP-DB and SFP-DB, respectively. After pretreatment, the yield of solid residues was defined as the mass ratio of pretreated DB to raw bamboo. The content of glucose, xylose and lignin in the solid residue are analyzed using the National Renewable Energy Laboratory (NREL) methods (Sluiter et al., 2008). The delignification, hemicellulose removal and glucose retention rate were calculated according to the following formula.

$$\text{Xylan removal}\left(\%\right)=1-\frac{\text{xylan}\;\text{in}\;\text{pretreated}\;\text{bamboo}\;\left(g\right)}{\text{xylan}\;\text{in}\;\text{the}\;\text{raw}\;\text{biomass}\;\left(g\right)}\times 100$$ (1)

$$\text{Delignification}\left(\%\right)=1\;-\frac{\text{lignin}\;\text{in}\;\text{pretreated}\;\text{bamboo}\;\left(g\right)}{\text{lignin}\;\text{in}\;\text{the}\;\text{raw}\;\text{biomass}\;\left(g\right)}\times 100$$ (2)

$$\text{Glucan recovery}\left(\%\right)=\frac{\text{glucan}\;\text{in}\;\text{pretreated}\;\text{bamboo}\;\left(g\right)}{\text{glucan}\;\text{in}\;\text{the}\;\text{raw}\;\text{biomass}\;\left(g\right)}\times 100$$ (3)

### 2.3 Enzymatic hydrolysis and ethanol fermentation

Enzymatic saccharification of pretreated samples were carried out in a shaking incubator using 50 mM sodium citrate buffer (pH = 4.8) at 50°C and 150 rpm for 72 h. Substrate concentration and cellulase dosage were 5% (w/v) and 20 FPU/g substrate. During the enzymatic saccharification, samples were taken at different times to monitor the hydrolysis progress. The glucose and xylose yield were determined using our previous methods (Jin et al., 2020; Meng et al., 2022).

The hydrolysate from the 72 h enzymatic hydrolysis was sterilized and added yeast inoculum of 10% (v/v). The flask was incubated in a rotary shaker at 35°C, 150 rpm for 24 h and samples were withdrawn periodically. The amounts of sugars and fermentation products were analyzed using HPLC (Agilent 1260 series, United States) equipped with an Aminex HPX-87H column. The mobile phase was 0.005 M H_2_SO_4_ at a flow rate of 0.6 mL/min. The yield of glucose and xylan, and ethanol were calculated according to the following formula.

$$\text{Glucose yield }\left(\%\right)=\frac{the\;weight\;of\;glucose\;in\;hydrolysate\times 0.9}{the\;weight\;of\;glucan\;in\;bamboo\;residue}\times 100$$ (4)

$$\text{Xylose yield }\left(\%\right)=\frac{the\;weight\;of\;xylose\;in\;hydrolysate\times 0.88}{the\;weight\;of\;xylan\;in\;bamboo\;residue}\times 100$$ (5)

$$\text{Ethanol yield }\left(\%\right)=\frac{ethanol\;concentration\;in\;fermentation\;broth\;}{glucose\;concentration\;in\;fermentation\;broth\times 0.51}\times 100$$ (6)

### 2.4 Cellulase adsorption of pretreated bamboo

The isotherms of enzymatic adsorption on the pretreated substrates were measured by mixing 1% (w/v) bamboo residues with Celllase 2730 (ranging from 0.01 to 0.8 mg/mL) at 4°C for 3 h at a stirring speed of 80 rpm. Centrifuged this mixture and analyzed the content of protein in the hydrolysate by Bradford assay. The amount of bound enzyme on the cellulose substrates was calculated by subtracting the amount of free enzyme in the hydrolysate from the initial enzyme. The obtained data were fitted based on Langmuir adsorption isotherm as described by Meng et al. (2022).

### 2.5 Analysis methods

The chemical structure and morphological features of samples was characterized by FT-IR spectrometer (Nicolet, United States) and SEM (Sigma 300, ZEISS, Germany). The hydrophobicity and enzyme accessibility of different pretreatment substrates was estimated by Rose Bengal dye and DR 28 absorption experiment using an ultraviolet-visible spectrophotometer (Tang et al., 2021). The degree of polymerization of different samples was measured by copper ethylenediamine method (Lin et al., 2020). XRD analysis was recorded by a D/MAX 2500PC diffractometer (Rigaku Corporation, Japan). Crystallinity index (CrI) was obtained by the ratio of the crystalline peak area to the crystalline peak and the amorphous peak area (Alam et al., 2020). Specific surface area was performed on Brunauer Emmett Teller (BET) assay with nitrogen gas adsorption (Micromeritics Instru-ment Corp., Norcross, United States). X-ray spectroscopy spectra were obtained with an Escalab 250Xi spectrometer (Thermo Fisher Scientific Inc., US), with nonmonochromatic Al K alph (15 kV, 300 W, 1486.8 eV). The surface coverage of lignin and carbohydrates on the surface of the samples before and after pretreatment was calculated as follows (Wan et al., 2023):

$$S_{\text{lignin}}\left(\%\right)=\frac{O/C_{\text{pretreated}}-O/C_{\text{carbohydrate}}}{O/C_{\text{lignin}}-O/C_{\text{carbohydrate}}}\times 100$$ (7)

$$S_{\text{carbohydrate}}=1-S_{\text{lignin}}$$ (8)

$$O/C_{\text{carbohydrate}}=0.83\;O/C_{\text{lignin}}=0.3$$

## 3 Results and discussion

### 3.1 Chemical compositions of bamboo after sequential sulfomethylation-Fenton oxidation pretreatment

**FIGURE 1:**
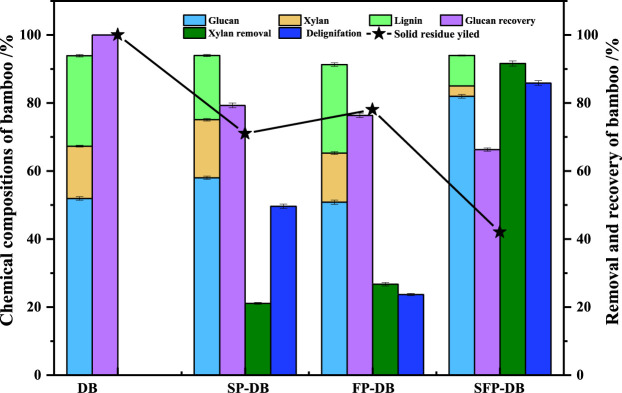
Effects of sequential sulfomethylation and Fenton oxidation pretreatment on the changes of bamboo residue composition.

The raw *D. branddisii* (DB) contained about 51.93% glucan, 15.37% xylan and 26.6% lignin, as presented in Figure 1. The solid recoveries of SP, FP and SFP process decreased to 71.2%, 78.6%, and 42.2%, respectively, which was due to the degradation and dissolution of lignin-derived compounds and hemicellulose-derived monosaccharides during the pretreatment process. It showed that the sulfomethylation reaction had a good auxiliary effect on Fenton pretreatment of DB. When raw material DB was treated with FP pretreatment alone, 26.72% of xylan were removed. The rest of the material consisted of 26.02% lignin, 14.44% xylan, and 50.83% glucan. Upon delignification using sulfomethylation reaction alone, an obvious decrease on lignin contents was observed from original 26.6%–18.86%, while there was no significant difference in xylan extraction. And it found that the solution of sulfomethylation pretreatment alone contained 1.14 g/L glucose, 0.33 g/L xylose and 1.5 g/L arabinose, while the liquid of Fenton pretreatment alone consisted of 0.303 g/L xylose, which was consistent with the changes in chemical composition in pretreated residues Previous studies have found that the sulfomethylation reaction can introduce sulfonic groups to the benzene ring of lignin, improve the lignin hydrophilicity, and ultimately lead to the lignin dissolution (Gu et al., 2013; Zhu et al., 2015). Surprisingly, the amount of xylan and lignin in DB decreased to 3.07% and 8.95% after the sequential sulfomethylation and Fenton oxidation pretreatment, with 85.87% and 91.61% of delignification and xylan removal rate, which was about 3.5 times higher than these of single FP-DB. Meanwhile, the liquid of the combined pretreatment contained 0.72 g/L glucose, 0.48 g/L xylose and 0.03 g/L arabinose. This predicted that the SFP-DB had a looser structure as well as higher enzymatic hydrolysis yield. It should be attributed to the partial removal of lignin during the sulfomethylation reaction, which can significantly increase the porosity of material. Then the delignification allowed the large hydroxyl radicals (HO and HOO·) generated by Fenton reaction to enter the cell wall of samples, greatly improving the removal rate of xylan and lignin in FP-DB substrates. Recently, some research focused on improving the performance of Fenton pretreatment, such as the release of more free radicals (Guzmán et al., 2015; Rafaqat et al., 2022) and the synergistic pretreatment using Fenton oxidation and other pretreatments (Li et al., 2016; Yu et al., 2017; Zhang et al., 2023). In addition, 66.29% glucan was retained in SFP-DB sample, indicating partial cellulose loss caused by the oxidative degradation under the severe acid and oxidative extraction conditions (Zhou et al., 2023), which was a major issue in the sequential sulfomethylation and Fenton oxidation pretreatment. Although cellulose was difficult to degrade under the sulfomethylation reaction, hydroxyl radicals from the Fenton reaction can depolymerize polysaccharides including glucan and xylan, thereby reducing the glucan content of Fenton pretreated DB (Hyde and Wood, 1997; Ray et al., 2010). The above results indicated that the combined pretreatment has a synergistic destructive effect on the natural recalcitrance of bamboo residues, which consequently enhanced the cellulose accessibility to enzymes.

### 3.2 Promotion of the sulfomethylation-Fenton pretreatment on enzymatic saccharification and ethanol production

**FIGURE 2:**
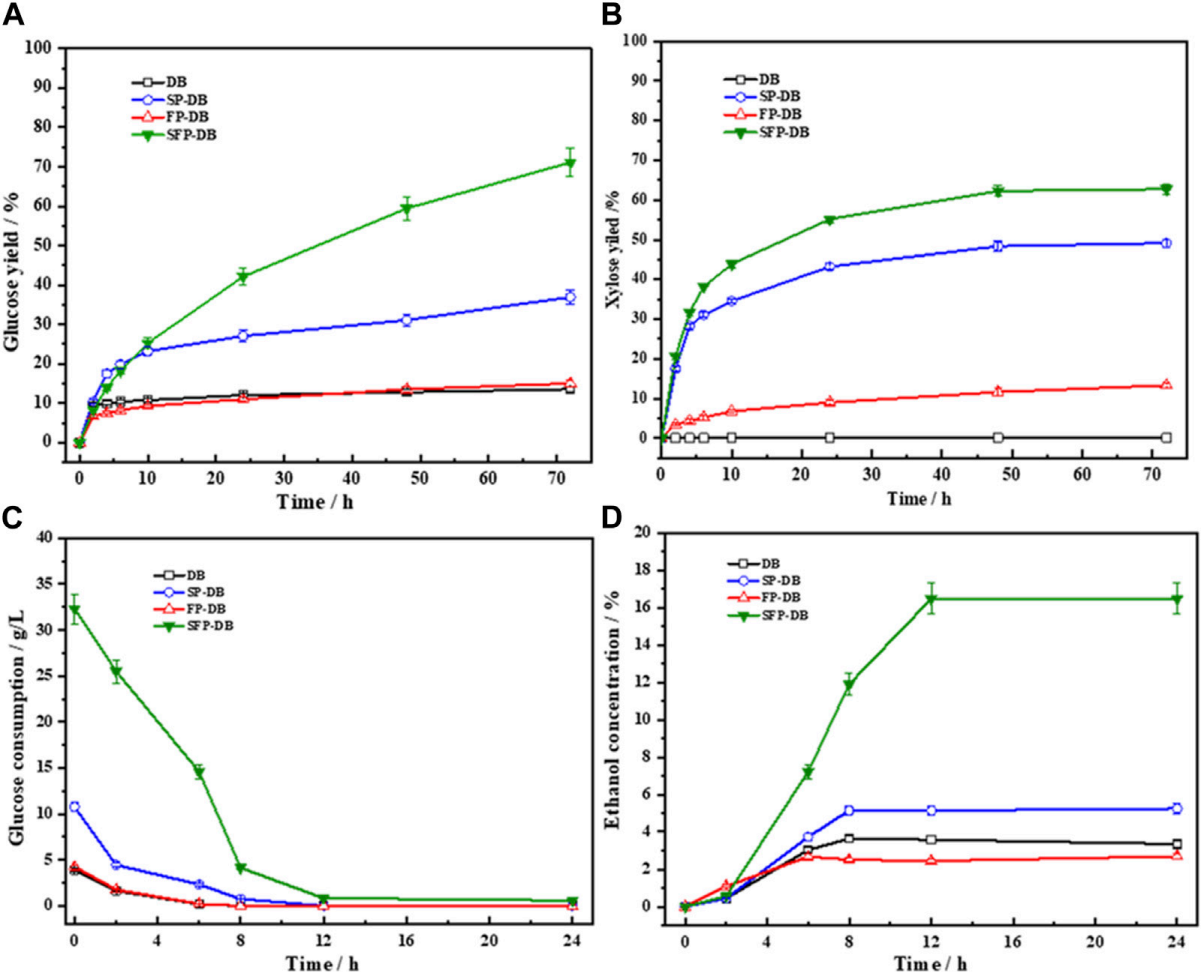
The glucose **(A)** and xylose yield **(B)** in the 72 h enzymatic hydrolysis of pretreated bamboo, as well as the glucose consumption **(C)** and ethanol production **(D)** of the hydrolysates during the subsequent fermentation process.

The pretreated substrates in this study were subjected to enzymatic saccharification to evaluate the pretreatment performance. Figure 2 clearly displays the improvement of enzymatic digestibility and ethanol production using the synergistic treatment. The glucose and xylose yield of raw DB were 12.09% and 10.3%, respectively. When DB was treated with Fenton oxidation reaction alone in the study, the yields of glucose and xylose (15.06% and 13.3% respectively) were not increased greatly, suggesting that Fenton reaction had an unsatisfactory pretreatment performance on bamboo residues. Compared with Fenton pretreated bamboo residues (FP-DB), the yields of glucose and xylose in the single sulfomethylation reaction were up to 36.97% and 49.13%, respectively increased by 2.5 and 3.7 times. Our previous studies have shown that the HO and HOO produced by Fenton reaction have high oxidative strength and can damage the structure of lignocellulosic biomass like microbial degradation (Wu et al., 2018; Zhang et al., 2023). However, we found that Fenton pretreatment performance of bamboo was limited due to its high degree lignification and extremely short half-life of hydroxyl radicals, which was also consistent with the results of Kato et al. (2014). Therefore, it is necessary to partially extract non-cellulose substances before Fenton oxidation pretreatment, providing broad space for bamboo Fenton reaction.

As expected, a significant increase in enzymatic hydrolysis was observed, when the sulfomethylation reaction was performed as a pre-step before Fenton oxidation pretreatment of bamboo. Specially, the yield of glucose reached to 71.11%, which was 4.72- and 1.92-folds higher than FP-DB and SP-DB, respectively. The xylose yield was enhanced from 13.3% of FP-DB and 49.13% of SP-DB to 67.84% of SFP-DB. The results again indicated that the Fenton pretreatment performance can be obviously improved by increasing the hydrophilicity of reaction substrates. Several studies have shown that Fenton pretreatment has great potential in the field of lignocellulose bioconversion, as long as sufficient hydroxyl radicals were provided (Kato et al., 2014; Jung et al., 2015; Li et al., 2016). In the pulp and paper industry, sulfomethylation reaction of biomass was often used to improve lignin hydrophilicity, finally dissolved lignin fraction. However, when bamboo residues were subjected to these two pretreatments alone, we found that the enzymatic digestibility efficiency of substrates were lower than 40%. In this study, faster saccharification rates and higher hydrolysis yield were obtained in the combined treatment of sulfomethylation and Fenton oxidation reaction. After being subjected to sulfomethylation, with hydrophilicity in samples increased, the compact bamboo structure became more looser, which greatly helped to the oxidative degradation of cell wall substances by hydroxyl radicals, including lignin, xylan as well as cellulose.

The hydrolysate obtained from 72 h enzymatic saccharification was subjected to ethanol fermentation after supplementation with necessary nutrients for yeast growth. It was found that the majority of glucose were consumed by *S. cerevisiae* within the initial 8 h (Figure 2C); the glucose concentrations in all liquids were finally lower than o.5 g/L after 12 h fermentation, indicating that the yeast could efficiently metabolize the glucose in the hydrolysate. The glucose consumption and ethanol content plateaued after 12 h fermentation. The maximum ethanol concentrations of 3.35, 2.69, 5.25, and 16.47 g/L were obtained for untreated DB, FP-DB, SP-DB and SFP-DB respectively, corresponding to the ethanol yields of 75.18%, 75.35%, 76.98%, and 74.68%. The SFP-DB substrate showed the highest sugar conversion rate and ethanol concentration, which was approximately 3–6 times higher than FP-DB and SP-DB, meant that the SFP process was an efficient pretreatment method of bamboo residues for the production of sugars and ethanol.

### 3.3 Effect of sulfomethylation-Fenton pretreatment on enzyme adsorption and cellulose accessibility of bamboo residues

**FIGURE 3:**
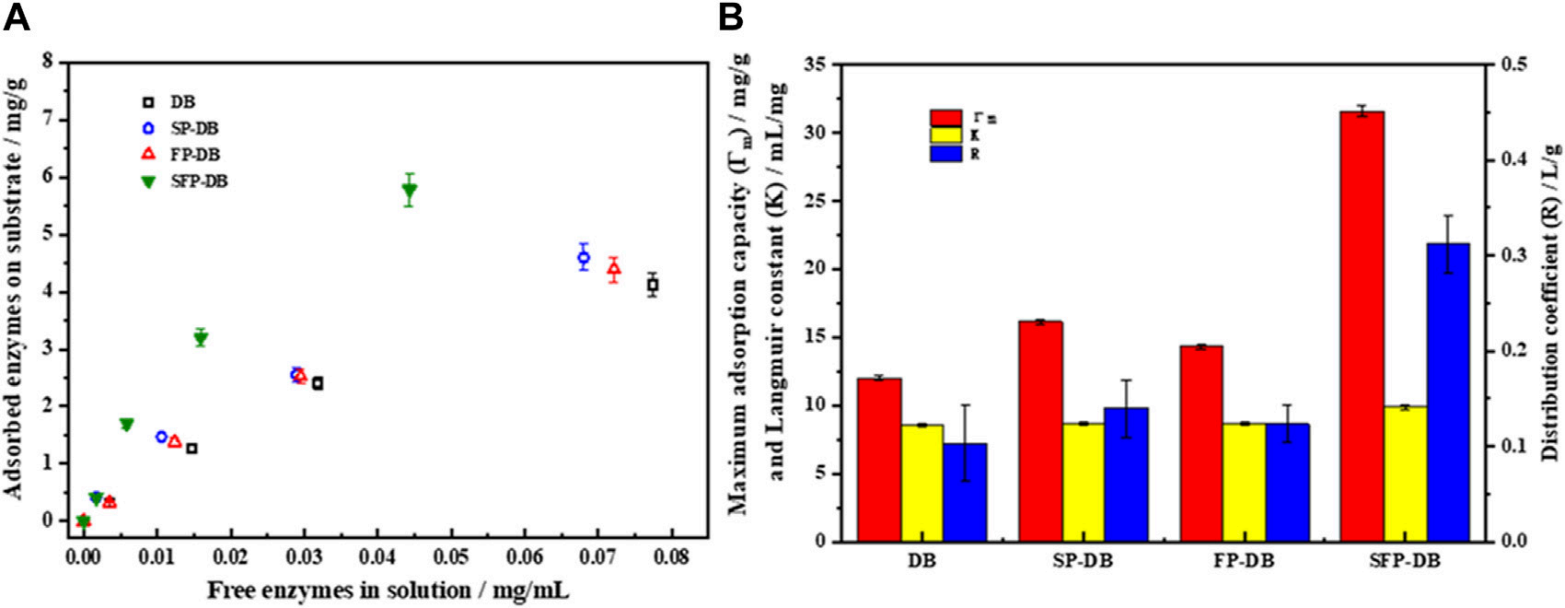
Cellulase adsorption isotherms **(A)** and Langmuir adsorption isotherm parameters **(B)** of pretreated samples.

As was well known, the amount of protein adsorbed on the substrate was considered a rate limiting step in the enzymatic saccharification. Cellulase adsorption capability on bamboo residues was studied using Langmuir adsorption isotherm. As shown in Figure 3, the cellulase affinity of untreated DB, FP-DB, and SP-DB was weak, and the maximum amount of adsorbed enzyme (Γ_max_) were 12.01, 14.32, and 16.14 mg/g sample, respectively. However, the maximum adsorption quantities of combined pretreatment (SFP) were significantly increased to 31.6 mg/g sample, improved by 2.2 folds than that on FP-DB and by 1.95 folds compared with SP-DB, suggesting that there existed numerous binding sites on the surface of SFP-DB than two single pretreated substrates. It can be clearly observed in Figure 3B that the Langmuir constants (K), as a parameter estimating the affinity of cellulase to substrate, was found to be 9.88 mL/g sample for SFP-DB, followed by 8.63 mL/g sample for FP-DB and 8.57 mL/g sample for SP-DB. Previous studies have shown that the maximum adsorption capacity was positively correlated with enzymatic hydrolysis efficiency, while there was no clear relationship between K value and enzymatic hydrolysis. This was due to the coexistence of productive adsorption of cellulose and non-productive adsorption of lignin on the substrate (Li et al., 2016; Mota et al., 2019). The distribution coefficient (R) was also used to quantify the amounts of cellulases onto substrates. The R value of SFP-DB (0.31 L/g) was about 2.5-folds higher compared with FP-DB and SP-DB. Additionally, the maximum adsorption capacity of pure cellulose and lignin with cellulase were 12.76 and 21.19 mg/g substrate, indicating that lignin has a higher adsorption capacity for cellulase compared to cellulose. After the combined pretreatment, the maximum adsorption capacity, affinity and distribution coefficient of pure cellulose were improved significantly 22.14 mg/g, 5.12 mL/mg and 0.11 due to the lignin removal. (date not shown). The above results meant that the interaction between SFP-DB and cellulase was more active and stronger in comparison with another two control samples. The remarkably higher adsorption capacity of SFP-DB should be attributed to its accessible cellulose surface areas, structural features of substrates in different pretreatments. The sulfomethylation and Fenton pretreatment can oxidize and degrade lignin, further improve hydrophilicity of pretreated substrates, thus decreasing the lignin content and the hydrophobicity of substrates. This will lead to the decrease of non-productive adsorption capacity between cellulases and lignin, while increasing the productive adsorption of enzyme on cellulose substrates.

**FIGURE 4:**
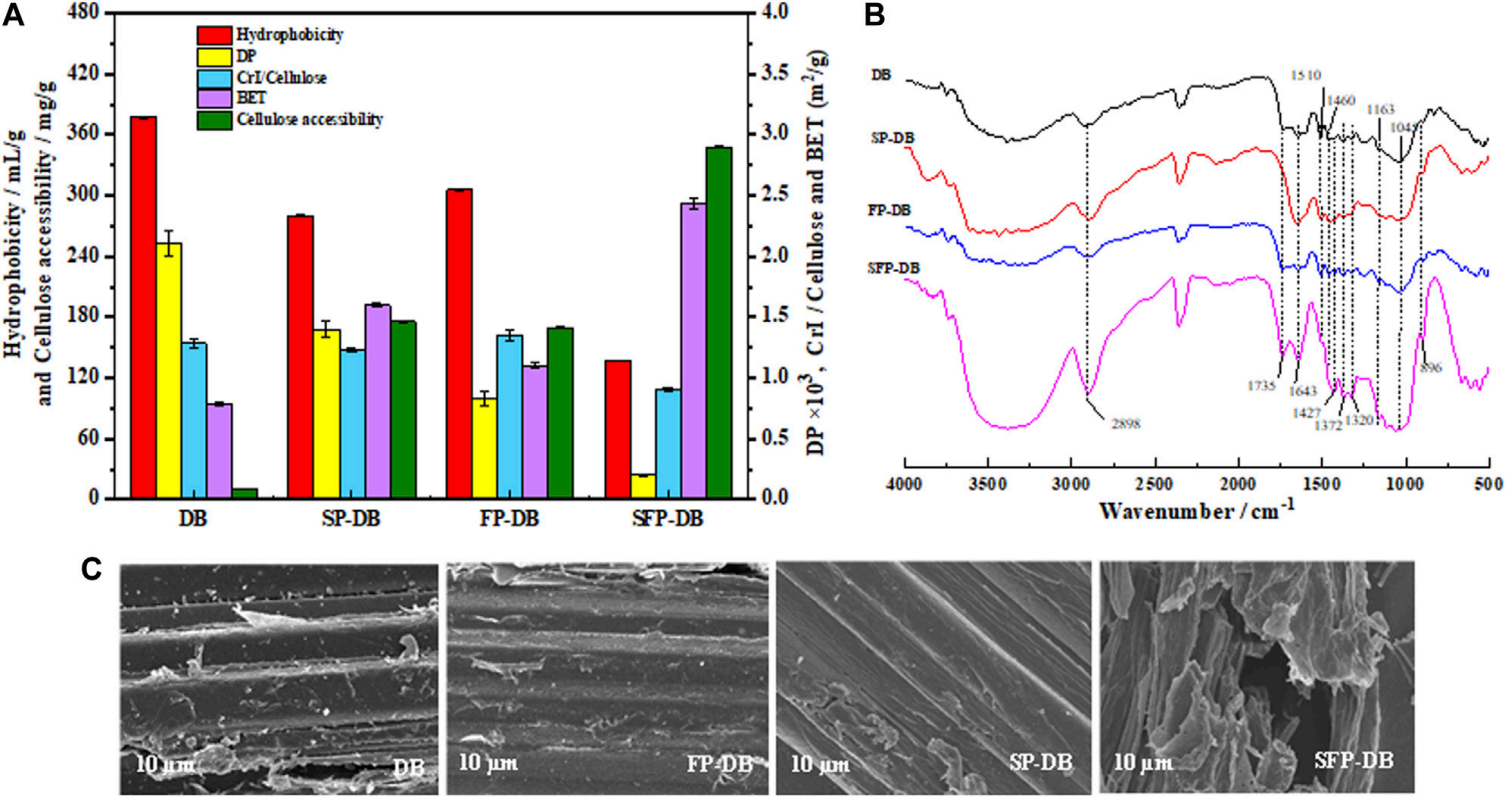
The physical properties **(A)**, FTIR spectra **(B)** and SEM **(C)** images of untreated and treated *D. branddisii*.

Cellulose accessibility was considered a key indicator and signal for improving the enzymatic saccharification efficiency (Arantes and Saddler, 2011; Rollin et al., 2011). The low enzymatic saccharification of untreated bamboo was believed to be because of ineffective lignin adsorption and poor cellulose accessibility. From Figure 4A, the accessibility of untreated DB was only 10.15 mg dye/(g material). It could be seen that the accessibility improved to 169.78 and 175.68 mg dye/(g material) when the single Fenton oxidation reaction and sulfomethylation were carried out. Using the sequential pretreatment, a further increase in accessibility (289.84 mg/g) was observed. This increase may be due to the lignin matrix as the main obstacle preventing enzymes from accessing cellulose substrate. When lignin was dissolved in large quantities, the accessibility and enzyme hydrolysis efficiency were improved. Another reason for the increasing accessibility was the improvement in hydrophobicity of SFP-DB, which reduced the nonproductive adsorption between pretreated samples and enzyme. Huang et al. (2022) concluded that there was a strongly positive correlation between enzyme digestibility efficiency and cellulose accessibility. Rollin et al. (2011) reported that the increase of cellulose accessibility was even more important than lignin removal for enhancing enzymatic hydrolysis. Therefore, the lignin sulfomethylation was a very effective method for improving the Fenton pretreatment efficiency of bamboo biomass.

### 3.4 Characterization of pretreated bamboo residues

Enzymatic saccharification is closely correlative to the physical and chemical characteristics of substrates, such as hydrophobicity, degree of polymerization (DP), crystallinity, and accessibility, as well as chemical structure of cellulose substrate. Figure 4 showed the change of these parameters after the different pretreatment. The hydrophobicity of raw DB, FP-DB and SP-DB substrates was determined to be 377.26, 305.64, and 280.27 mL/g substrate, respectively. Compared with FP-DB and SP-DB, the hydrophobicity of SFP-DB (137.44 mL/g substrate) was further reduced by about 2 times. The decrease was mainly attributed to the removal of amorphous components and the introduce of sulfonic acid groups in the substrates structure during the combined pretreatment process. The decrease of hydrophobicity can reduce the nonproductive bind of cellulase to the pretreated samples. The CrI of the raw and treated substrates was measured using XRD technology. As seen from Figure 4A, the CrI/cellulose content was obviously decreased from 1.35 for FP-DB and 1.24 for SP-DB to 0.9 for SFP-DB, suggesting that not only amorphous substances were removed, but the crystalline region also could be opened during the sulfomethylation-assisted Fenton oxidation pretreatment of bamboo biomass, as also revealed by the compositional analysis of samples (Figure 1). The lower crystallinity/cellulose value of SFP-DB, making cellulose substrates easily damaged by cellulases, was significantly related to the improvement of enzymatic digestibility (Ling et al., 2017). Degree of polymerization (DP) is another important property of cellulose. The degree of polymerization was 2111 for the original material. After pretreatment, the DP showed a significant downward trend. The DP of FP and SP were 1399 and 831, respectively, which are 1.5–2.5 times lower than the original sample. This indicates that the single sulfomethylation and Fenton oxidation reaction both resulted in the hydrogen bonds breakage and the scission of cellulose chain in lignocellulosic biomass, and generated the shorter cellulose chains, which was beneficial to the subsequent saccharification process. The DP of SFP-DB (201) followed a similar decreasing trend, which was 4.1–6.9 folds lower than the DP of the two single pretreatment, indicating the generation of more cellulose reducing end. Certainly, DP was not typically an independent parameter influencing cellulose hydrolysis because the change of DP was often accompanied by changes of crystallinity.

The specific surface area and pore features of pretreated bamboo were studied using BET analysis with nitrogen gas adsorption. Porous structure usually implied a larger specific surface area of cellulose was more susceptible to bind with cellulase enzyme (Ma et al., 2021). The specific surface area of native DB was determined to be 0.78 m^2^/g. Subsequently, the BET values using the single sulfomethylation and Fenton oxidation reaction were 1.6012 m^2^/g and 1.104 m^2^/g, respectively, while the combined pretreatment resulted in a specific surface area of 1.9375 m^2^/g. Compared with the FP-DB sample, the specific surface area of SFP-DB increased by 75.5%. The highly disordered morphological structure upon the disruption of the lignin and hemicellulose layer during the combined pretreatment can result in an improvement in the specific surface area of substrates (New et al., 2021). SEM analysis used to visualize the changes of lignocellulose surface structure. As seen in the SEM image (Figure 4C), original DB surface was smooth and dense morphology, which can hinder the cellulose accessibility to enzymes. After the sequential pretreatment of sulfomethylation and Fenton oxidation reactions, the substrate surface becomes rough, creating more pores and increasing the exposure of fiber surface reaction sites, which would significantly increase the cellulose accessibility and facilitate the enzymatic saccharification.

The surface lignin coverage as well as surface chemical bonds were explored using XPS analysis (Table 1). The O/C ratio of raw DB was 44.34% and surface lignin coverage was 77.32% in Table 1. When DB was treated with the sulfomethylation extraction at 70°C for 1 h, the O/C ratio and surface lignin coverage decreased to 47.43% and 71.15%, respectively because of lignin dissolution and cellulose exposure. Interestingly, FP-DB showed a small increase in content of surface lignin (82.55%), which is thought to be due to partial lignin melts and repositions on the surface under harsh oxidation conditions (Hu et al., 2013). This phenomenon caused cellulase to irreversibly bind to lignin on the substrate surface. As expected, SFP-DB had the lowest surface lignin coverage (52.62%) and the highest O/C ratio (56.69%), meant that the combined pretreatment contributed to decreasing and redistributing the surface lignin, which resulted in a higher extent of delignification as well as enzymatic saccharification yield on SFP-DB substrates compared to the other samples. As shown in Table 1, high-resolution carbon spectrum of C1s exhibited four types of functional groups, with binding energies of 284.60 eV (C-C/C-H), 286.10 eV (C-O), 287.40 eV (C-O-C), and 289 eV (O=C-O), respectively. The relative content of C–C/C–H on the surface of pretreated substrates increased from 37.37% for raw DB to 42.97% for FP-DB and 38.98% for SP-DB. Meanwhile, the relative contents of C-O and C-O-C on the surface of the two single pretreated samples decreased. However, the opposite phenomenon was observed in SFP-DB. The relative content of C–C/C–H on the surface of SFP-DB decreased to 33.33%, while that of C–O and increased to 51.03%, indicating the increase of the exposed cellulose and surface hydrophilicity in SFP-DB.

**TABLE 1:** The surface lignin coverage and chemical properties of pretreated substrates by XPS analysis.

Samples	O/C ratio (%)	Surface lignin coverage (%)	Surface carbohydrate coverage (%)	Surface chemical bonds (%)			
				C-C, C-H (284.6, eV)	C-O (286.1, eV)	C=O (287.4, eV)	O-C-O (289, eV)
*D. brandisii*	44.34	77.32	22.68	37.37	37.09	13.57	11.97
SP-DB	47.43	71.15	28.85	38.98	36.55	14.19	10.29
FP-DB	41.72	82.55	17.45	42.97	30.39	12.41	14.23
SFP-DB	56.69	52.62	47.38	33.33	51.03	6.48	9.17

Additionally, FTIR is used to study the changes in chemical functional groups present in the original DB and pretreated samples. As seen in the FTIR image of Figure 4B, the 3426 cm^−1^ peak, as the stretching vibration of –OH (Kăcuŕaková et al., 2000). Due to the lignin removal by pretreatment process, the hydrophobicity of pretreated samples decreased, led to the appearance of more hydrophilic -OH. As a result, the peak of SFP-DB at 3426cm^−1^ became wider and stronger. The decrease of absorbance at 1735 cm^−1^ after SFP indicate that the acetyl group in the hemicellulose region of bamboo residues has been removed. At 1602 and 1510 cm^−1^, corresponding to the aromatic ring stretching, the absorption peak of SFP-DB weakened and almost disappeared due to the removal of lignin. It could be observed that the intensity of 1043 cm^−1^ belonged to the C-O-C vibrations of pyranose ring skeleton in cellulose and 900 cm^−1^ from the β-glycosidic linkages was obviously increased, which might be related to the increase of cellulose content in SFP-DB sample. As a result, all these changes generated by the combined pretreatment will inevitably increase the accessibility between biomass and cellulases, thereby promoting the conversion of biomass into fermentable sugars.

### 3.5 Mass balance of the sequence sulfomethylation and Fenton oxidation pretreatment

**FIGURE 5:**
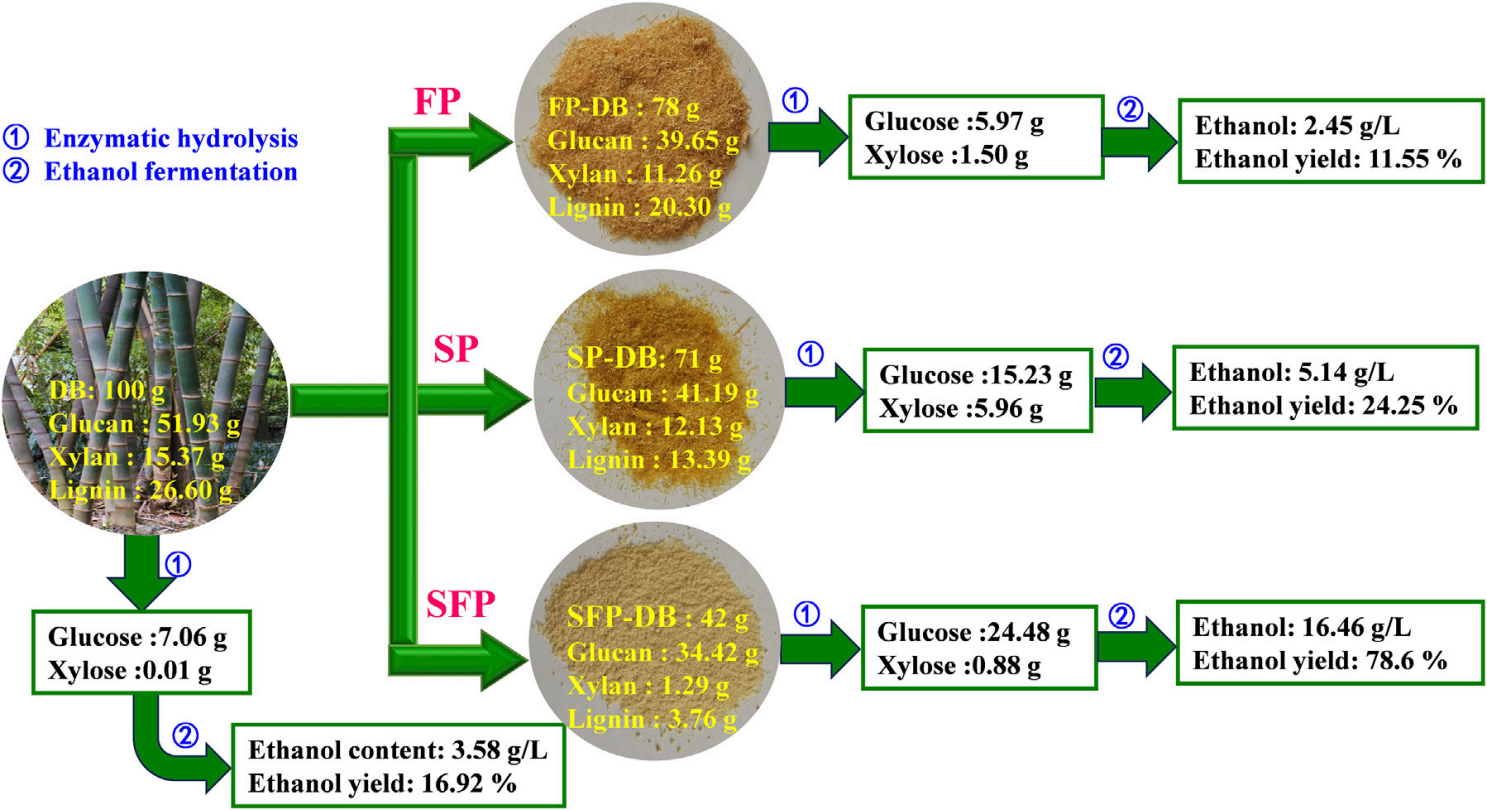
Material balance of the sulfomethylation-Fenton pretreatment, followed by enzymatic hydrolysis and fermentation processes.

Figure 5 shows our researches on the recovery of glucose and xylose, and ethanol production from *D. branddisii* using different Fenton oxidation pretreatment. 100 g untreated DB produced 7.06 g glucose, 0.01 g xylose and 3.58 g/L ethanol with 16.92% ethanol yield. After the Fenton pretreatment alone, 78 g solid substance was obtained, which were mainly composed of glucan (39.65 g), xylan (11.26 g) and lignin (20.3 g). Then FP-DB were hydrolyzed by cellulase (20 FPU/g sample) and produced about 5.97 g glucose and 1.5 g xylose. Subsequently, a low content of ethanol (2.45 g/L) was observed. However, when treating DB through the integrated sulfomethylation and Fenton oxidation pretreatment, 42 g of residues were left. The remaining residues consisted of 34.42 g of glucan, 1.29 g of xylan, and 3.76 g of lignin. After the enzymatic saccharification, the yield of glucose reached to 24.48 g, improved by about 4.1 times higher than that of FP-DB. The highest ethanol content of the hydrolysate can reach 16.46 g/L, with a fermentation efficiency of 78.6%, increased by 6.7-folds than that of FP-DB.

## 4 Conclusion

The combination of sulfomethylation and Fenton oxidation pretreatment (SFP) was proved to achieve an easily digestible material resulting in efficient enzymatic hydrolysis efficiency and ethanol production. The SFP-DB resulted in 71.11% glucan conversion, 67.84% xylan conversion, and a total sugar yield of 24.48 g per 100 g dry bamboo. After 24 h of fermentation, 16.46 g/L of ethanol was found. The SFP induced the changes of physiochemical characterization in bamboo biomass, fully exposed cellulose, porosity structure, the increase of hydrophilicity, the disruption of polymerization degree and crystalline structure, which facilitated the bioconversion to fermentable sugars and ethanol.

## Data Availability

The raw data supporting the conclusion of this article will be made available by the authors, without undue reservation.
